# Fatigue assessment for back-support exoskeletons during repetitive lifting tasks

**DOI:** 10.3389/fbioe.2024.1418775

**Published:** 2024-09-25

**Authors:** Xiaohan Xiang, Masahiro Tanaka, Satoru Umeno, Yutaka Kikuchi, Yoshihiko Kobayashi

**Affiliations:** Institute of Agricultural Machinery, National Agriculture and Food Research Organization (NARO), Saitama, Japan

**Keywords:** low back pain, safety assessment, exoskeleton, lumbar fatigue, human simulation

## Abstract

Fatigue is a major cause of low back pain for workers in various fields, including industry and agriculture. It has a negative impact on workers’ safety, decreases their productivity, and causes a reduction in their occupational career. An exoskeleton is expected to be a solution for reducing workers’ fatigue. However, assessing the safety and effectiveness of exoskeletons, except for the direct measurement of electromyography (EMG) in the human body, is challenging in real-case scenarios. Recently, simulations have been widely used to estimate biomechanical variables. Thus, we aimed to develop a method that combines an exoskeleton model and human body simulation to evaluate the effects of exoskeletons on lumbar fatigue. The strength and tendency estimated using this method are similar to those obtained from EMG devices in symmetrical repetitive lifting tasks. In addition, this method can be used to predict and simulate fatigue after a recorded motion. Our findings will help guide manufacturers in designing their products.

## 1 Introduction

Low back pain (LBP) commonly occurs in workers in the rehabilitation, industry, and agriculture fields because they frequently perform manual handling tasks with heavy loads, awkward postures, and repetitive movements (Rosecrance et al., 2006; Fathallah et al., 2008; Du et al., 2022). There is a growing shortage of workers in elderly societies. Thus, it is necessary to protect workers’ lumbar spines and increase their career periods. A lumbar-type exoskeleton provides assistive torque and is expected to protect the lumbar (Upasani et al., 2019). Validating exoskeletons’ safety is necessary to guide users in appropriately selecting suitable exoskeletons and using them.

Many studies have demonstrated the effectiveness of exoskeletons in protecting the lumbar spine by reducing the peak lumbar load at the L5/S1 level (the fifth segment of the lumbar spine and first segment of the sacrum) (Weston et al., 2018; Koopman et al., 2019; Marras et al., 1999; Abdoli-e and Stevenson, 2008). In repetitive tasks, other than the peak lumbar load, lumbar fatigue is a major contributor to LBP (Waters et al., 1993). As the repetition increases, fatigue accumulates in the lumbar system, muscle contraction is affected, and the muscle strength affects the fatigue levels (Gallagher and Schall, 2020). The effectiveness of exoskeletons for repetitive tasks has been reported (Omoniyi et al., 2020). However, the effect of using an exoskeleton on lumbar fatigue has not been quantitatively evaluated.

The state-of-the-art exoskeleton assessment of lumbar fatigue is based on a comparison between biomedical measurements before and after repetitive tasks, such as the maximal voluntary contraction (MVC) and median frequency of the trunk muscles from electromyography (EMG) recordings, heart rate, or oxygen consumption, to estimate the relief of lumbar fatigue (Godwin et al., 2009; Ulrey and Fathallah, 2013; Poliero et al., 2020; Madinei et al., 2020; Whitfield et al., 2014; Xiong et al., 2019). However, this method of estimating strength can be affected by individual differences (Godwin et al., 2009) and measurement errors (Stålberg et al., 2019), making it difficult to quantify the effect of the exoskeleton. In addition, because the measurements are obtained from the human body considering an acceptable fatigue of the subjects, the current exoskeleton assessment method cannot predict the effect of the exoskeleton on a long repetitive motion.

Simulations provide a new method to assess the exoskeleton that does not have to be based on biomedical measurements for every task. For example, a biomechanical model, which is widely used for estimating the lumbar load, can overcome the difficulty of obtaining biomedical measurements. Recent studies have shown that fatigue can be estimated based on human motion and joint loads (Dode et al., 2016; Jaber et al., 2013; Ma et al., 2009; Calzavara et al., 2019). Conversely to the biomedical measurements, motion and lumbar load can be estimated using optical devices, inertial measurement unit (IMU) systems, and biomechanical models (Lorenzini et al., 2019; Peternel et al., 2016; Gallagher et al., 2017; Zelik et al., 2022). In addition, functional analysis facilitates the prediction of long repetitive motions from a short period of experimental data. It is designed to handle functional data, such as body positions and trunk angles, accounting for their continuous nature and temporal dependencies (Ramsay and Silverman, 2005). Functional analysis has been used to accurately estimate continuous growth tendencies and demonstrate significant differences in the fatigue-induced kinematic changes (Xu et al., 2018; Godwin et al., 2010). Moreover, machines or humanoids can replace humans in testing the assistive torque using exoskeletons (Nabeshima et al., 2018; Ito et al., 2018). However, inaccurate conclusions can be drawn when extrapolating the exoskeleton results obtained using machines or humanoids to humans.

To estimate the effect of the exoskeleton on lumbar fatigue, we considered the development of a new fatigue assessment method that could overcome the shortcomings of traditional biomedical measurements. The novelty of this study is the development of a fatigue assessment method that combines an exoskeleton model, functional analysis, and biomechanical simulation to provide a quantitative assessment of various exoskeletons, which can reduce the individual differences and recording error from biomedical signals, and to predict the exoskeleton effect by predicting the afterward motion.

We aimed to develop a fatigue assessment method to evaluate the effects of exoskeletons on lumbar fatigue. Short periods of human motion data were recorded using this method. Long-term repetitive human motion data can be estimated based on Fourier functions that fit short-term motion data (Ramsay and Silverman, 2005). The assistive torque of the exoskeleton was estimated using a machine platform (Xiang et al., 2023). The exoskeleton’s characteristic curves, which present the relationship between the assistive torque, trunk angle, and trunk angular velocity, were also obtained. Subsequently, the combined model with an exoskeleton and human body could estimate the lumbar torque (with and without the exoskeleton’s assistive torque). Finally, we obtained the muscle strength using a fatigue model with the estimated motion and lumbar torque. Thus, the effect of the exoskeleton on the lumbar strength can be estimated without using EMG data. The subject is prevented from participating in long-term fatigue testing in actual tests.

## 2 Methods

**FIGURE 1:**
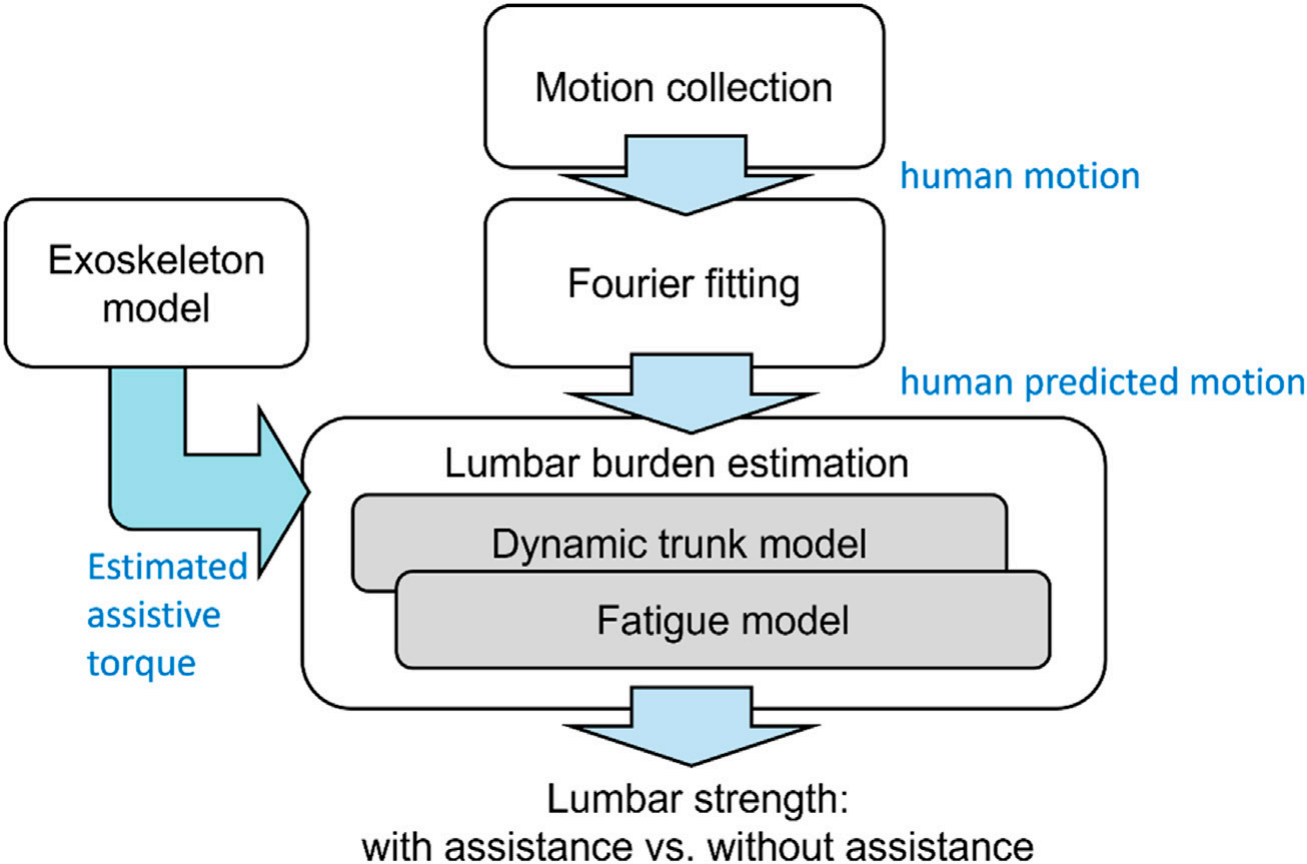
Procedure of fatigue assessment method.

A fatigue assessment method is proposed, as shown in Figure 1. The approach employed human trunk and fatigue models to estimate the lumbar torque and fatigue, respectively. Furthermore, the exoskeleton model introduced by Xiang et al. (2023) was used to compute the assistive torque, whereas the model’s fitting data (original assistive torque) was obtained using a testing platform, whose structure was introduced by Tanaka et al. (2020). The input for the exoskeleton model was the motion derived from a human lifting simulation, obtained using Fourier series equations.

### 2.1 Participants and tasks

Eleven male participants (height: 1.69 ± 0.06 m, body mass: 62.6 ± 12 kg, age: 24.0 ± 4.2 years) provided written consent to this experiment. Because repetitive lifting poses a high risk of LBP, younger subjects can tolerate relatively high lumbar loads. This study was approved by the ethics committee of the National Agriculture and Food Research Organization (approval no. Kakushin-ken_Rinri_22-30).

**FIGURE 2:**
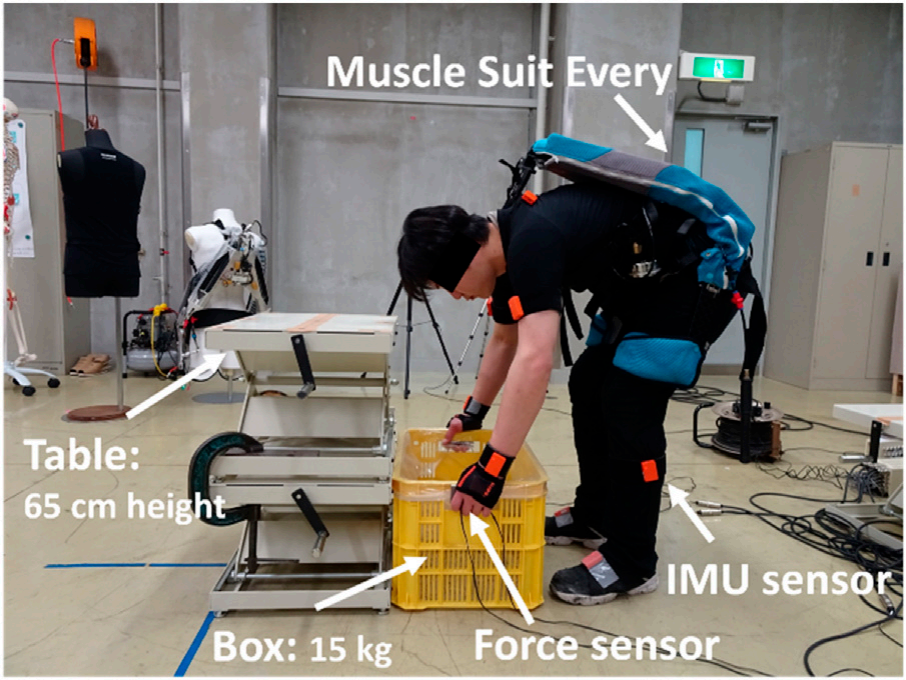
Repetitive lifting task of a 15 kg box with an exoskeleton. The subject conducted only 15-kg box liftings from the ground to the table repetitively; the return of the box from the table to the ground was done by other people.

As shown in Figure 2, each subject performed a symmetric repetitive lifting task from the ground to a 65 cm table. This task simulated a fertilizer-lifting task from the ground to the rear of a mini-truck. First, each subject performed the repetitive lifting task without an exoskeleton and then performed the same task with an exoskeleton. The interval between the tasks with and without an exoskeleton was 20 min to allow the subject to recover. For each condition, the repetitive lifting task consisted of 35 lifts, and the interval between two lifts was 8 s. Before and after the completion of the entire repetitive lifting task in each condition, the MVC of the four back muscles (the left/right thoracic spinae and left/right multifidus) was determined. We attached the surface electrodes by palpating the subjects and followed the suggestions from previous references considering that the thoracic erector spinae are 5 cm lateral to the T9 spinous process (McGill, 1992) and the center-to-center line is between two electrodes along the muscle fiber. The multifidus muscle setting was based on Toshiya (2010): the electrodes were placed between the L5 level and the upper iliac crest side end (2–3 cm from the spinal midline) along the connecting line between the upper iliac crest side end and spinous process of L2. The MVC testing method was taken from McGill (1991) and Toshiya (2010). The human trunk muscles play different roles in body motions. Erector spinae and multifidus muscles are selected in this study because they play an important role in symmetric lifting motion (forward flexion-extension) as reported by Bogduk (2005). Before the experiment, the participants were instructed to perform manual handling tasks at their preferred speeds to test their strength.

### 2.2 Instrumentation

As shown in Figure 2, a motion capture system (Xsens MVN Analyze, Xsens, Inc., Enschede, Netherlands) was used to reconstruct the whole-body motion. Four three-axis force sensors (USL08-H6; Tec Gihan Co. Ltd., Kyoto, Japan) were used to record the external loads acting on the human body. The box size was 57 × 28 × 10 cm, with a total mass of 15 kg. EMG sensors (MQ16, Kissei Comtec, Inc., Nagano, Japan) were attached to the subject’s back to obtain the MVC and continuous EMG data. The data recorded at 60 Hz were filtered using a low-pass filter with a 4 Hz cut-off frequency. An exoskeleton product called ‘Muscle Suit Every’ (MSE) (Innophys Inc., Tokyo, Japan) was used in this study. The mass of this MSE is 3.8 kg, the maximal assistive torque can be 100 N·m, and the permitted temperature is between −30°C and 50°C. In practical applications, the recommended air pressure pumped in the exoskeleton is approximately 0.1 MPa, which will provide much lower assistive torque than the maximal one. The mechanism of this device is as follows: When the user lifts a load, two McKibben muscles on the left and right sides exert contraction forces by air pressure. The lumbar moment is compensated by the torque supplied by the contraction force. In this study, the body motion parameters with and without the exoskeleton are measured during the first *T* period as shown in Figure 2.

### 2.3 Fourier basis function fitting repetitive motion data

**FIGURE 3:**
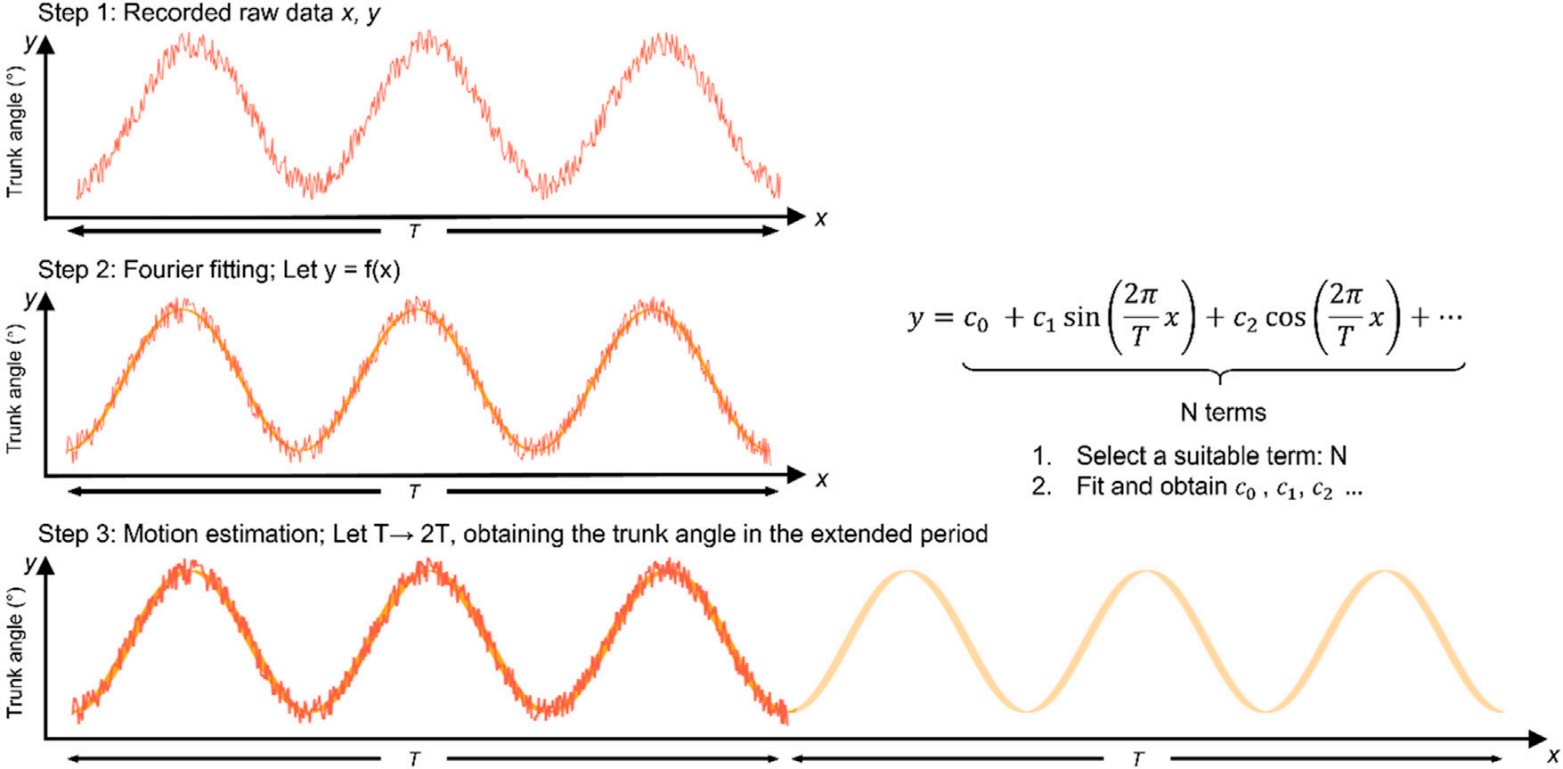
Schematic of motion data with Fourier basis function fitting. The motion was recorded in the experiment time *T* in step 1; then, the recorded motion was fitted by the Fourier basis functions in step 2; finally, the obtained Fourier basis functions created the afterwards motion (from *T* to 2*T*) in step 3.

To reduce the fatigue risk to the subjects, the motion after 5 min was simulated by the Fourier basis function fitting data using the previous 5 min of motion. The analysis process is illustrated in Figure 3. The biomechanical time-series discrete data on the trunk angle, angular velocity, and angular acceleration were converted to functional data for the functional analysis using Equation 1, which can be expressed as follows:

$$y_{i}\left(t\right)=\sum_{n=1}^{N}c_{in}\varphi_{n}\left(t\right)$$ (1)

where $y_{i}\left(t\right)$ represents the function converted from the *i*-th observed data series; *t* represents the number of time points; $c_{in}$ represents the coefficients; and $\varphi_{n}\left(t\right)$ are the Fourier basis functions with the number, *N*. Subsequently, the residual sum of squares and a penalty term based on the second derivative of the fitted curve were minimized (Ramsay and Silverman, 2005). This study assumes that the lifting movement does not change with fatigue.

**FIGURE 4:**
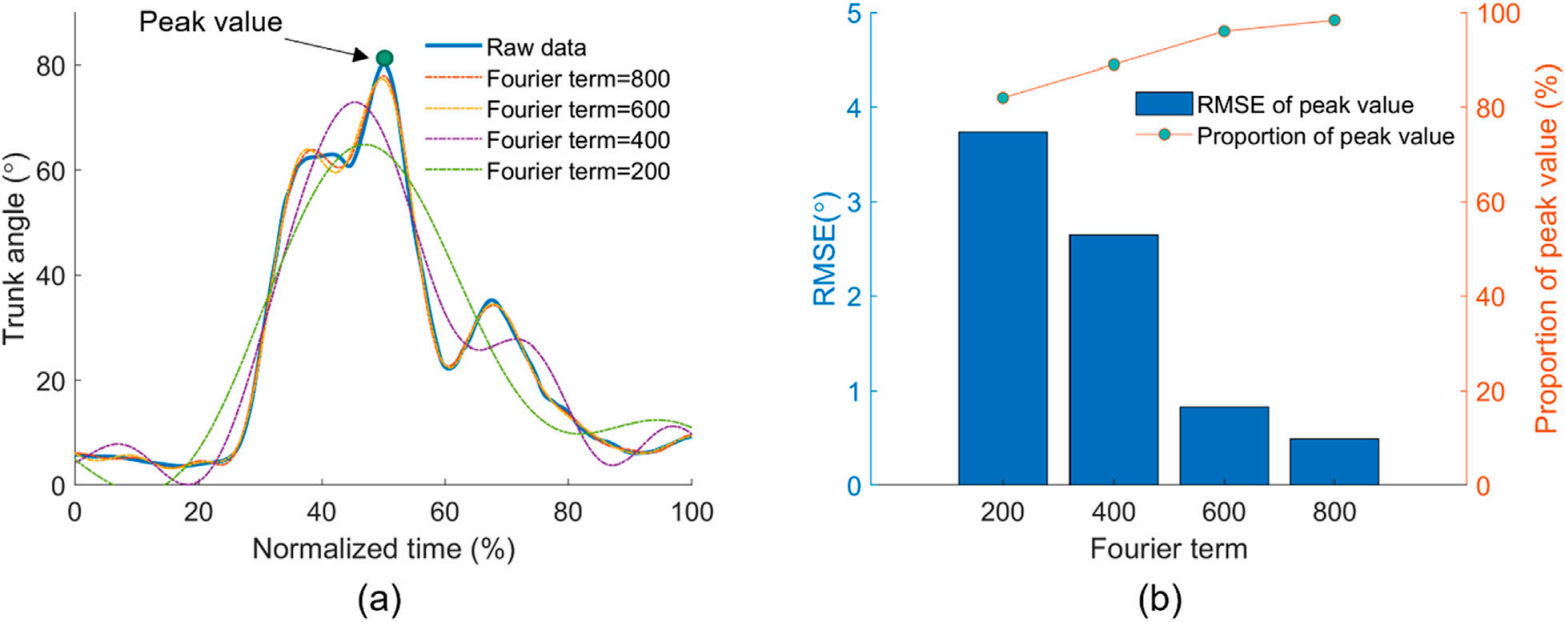
Selection of Fourier basis function terms *N*. **(A)** Comparison between the recorded trunk angle and estimated trunk angle by Fourier fitting functions with different terms in one lifting trial. **(B)** RMSE and accuracy between the recorded and estimated peak trunk angles from different Fourier terms.

The Fourier fitting results are presented in Figure 4. In Figure 4A, only Fourier terms larger than 600 fit the tendency well for the representative lifting sample. The root mean square error and proportion of the estimated peak trunk angle to the measured peak trunk angle from all trials are shown in Figure 4B. As the term increased from 200 to 800, the proportion increased from 82% to 98%, and the root mean square error (RMSE) reduced from 3.74 to 0.49. In this study, 800 was selected as the Fourier term *N*.

### 2.4 Lumbar load estimation (human and exoskeleton models)

**FIGURE 5:**
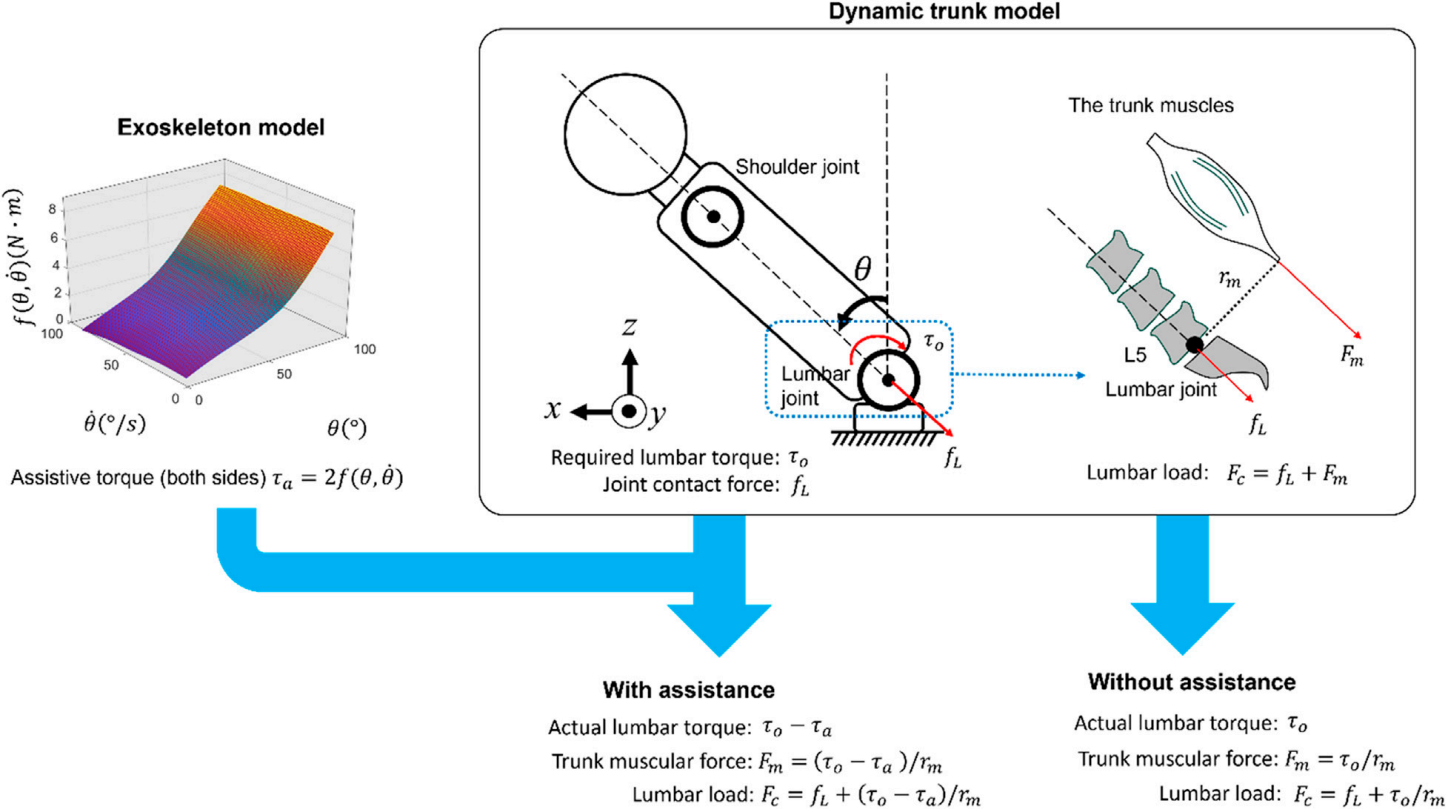
Estimation of actual lumbar torque and lumbar load using human and exoskeleton models; the assistive torque of the exoskeleton was estimated by the trunk angle θ and the trunk angular velocity $\dot{\theta }$ . The relationship between the assistive torque, trunk angle, and angular velocity was established by the thin-plate spline interpolation method, which uses the tested characteristic curves from the machine reported by Tanaka et al. (2020). The required lumbar torque $\tau_{o}$ was estimated using the dynamic trunk model. The actual lumbar torque and lumbar load changes depending on whether the lumbar was assisted.

In Figure 5, the lumbar torque (left side) was estimated using a trunk model similar to that reported previously. During the lifting task, the load was assumed to be attached to the shoulder joints (Nabeshima et al., 2018). The body parameters were calculated based on the body height and mass, and inverse dynamics were used to compute the lumbar torque $\tau_{o}$ in MATLAB (version 2023a) (Thomas et al., 2022). The assistive torque from the exoskeleton was estimated using the trunk angle and trunk angular velocity using the thin-plate spline interpolation method. The parameters of the interpolation method were determined by the raw data obtained from a machine measuring the assistive torque as the exoskeleton’s joint angle changed under different angular velocities. (Xiang et al., 2023; Tanaka et al., 2020). To obtain the actual torque at the lumbar joint, the assistive torque in this plane was subtracted from the non-assisted torque. The trunk muscular force ( $F_{m}$ ) was calculated using the lumbar torque divided by the representative trunk muscular moment arm length for a symmetric lifting task (Chaffin et al., 2006). Finally, the lumbar load was obtained using the force resulting from the muscular forces and the upper body load in the direction perpendicular to the lumbar vertebra using Equation 2, which was calculated as follows:

$$F_{c}=f_{L}+F_{m}=\left\{\begin{array}{l} f_{L}+\frac{\tau_{o}}{r_{m}},without\;assistance\\ f_{L}+\frac{\left(\tau_{o}-\tau_{a}\right)}{r_{m}},with\;assistance \end{array}\right.$$ (2)

where $F_{c}$ is the lumbar load (N); $f_{L}$ is the joint contact force (N), which is the upper body load acting at the fifth lumbar vertebra (L5) level and acting along the direction perpendicular to the crossing-section of the L5 level; $\tau_{o}$ is the L5 required lumbar torque (N·m); $\tau_{a}$ is the total assistive torque by the exoskeleton; and $r_{m}$ is the representative moment arm of the trunk muscles related to the L5 level, $r_{m}$ = 0.05 m (Chaffin et al., 2006). The actual lumbar torque is exerted by the trunk muscular force ( $F_{m}$ ) with the moment arm $r_{m}$ . Without assistance, the actual lumbar torque equals the required lumbar torque $\tau_{o}$ ; with assistance, the actual lumbar torque is the required lumbar torque minus the total assistive torque $\left(\tau_{o}-\tau_{a}\right)$ .

### 2.5 Fatigue model

As the subject performs a lifting task, the lumbar region is fatigued, and the current fatigue condition can be estimated based on the actual lumbar torque and initial fatigue condition. The joint strength S at $t_{1}$ can be calculated using Equation 3, which can be expressed as follows (Ma et al., 2009):

$$S\left(t_{1}\right)=S\left(t_{0}\right)e^{-\frac{k}{S_{\max}}\int_{t_{0}}^{t_{1}}\tau \left(t\right)dt}$$ (3)

where $S\left(t_{0}\right)$ is the initial maximal lumbar joint strength at $t_{0}$ , $S_{\max}$ is the maximal original lumbar joint strength, and $\int_{t_{0}}^{t_{1}}\tau \left(t\right)dt$ is the accumulated actual lumbar torque. We used k = 0.5 min^−1^, the average value estimated from all the back/hip models (Ma et al., 2011), and $S_{\max}$ = 212 N·m, the mean value of the maximal strength between 0° and 90° flexion (Chaffin et al., 2006).

As the subject rests between the two tasks, the lumbar spine is in the recovery process and finally recovers to the original maximal strength. The joint strength S can also be obtained using the recovery model (Liu et al., 2002). In this process, we only considered the effect of the exoskeleton on the fatigue condition; the recovery process is not presented in this study.

### 2.6 Statistics

The strength after the completion of each task was normalized to the initial strength. The average MVC of all measured trunk muscles was taken as the current lumbar strength. The strength obtained using the exoskeleton model was estimated based on the motion and lumbar load in the time history. The Mann–Whitney test was used to reveal if there were differences between the lumbar strength estimated by the fatigue assessment method and the MVC testing. The peak and average values of the body parameters (trunk angle and horizontal distance between the lumbar spine and wrist) and lumbar load were compared with and without the exoskeleton in the paired t-test. To evaluate the effectiveness of using an exoskeleton on the strength in repetitive lifting, a paired t-test was used to investigate the simulated longer-level fatigue from period *T* to period 2*T*. The estimated MVC before and after each task was used to obtain a regression model $S=ae^{bt}$ , where the coefficients *a* and *b* were determined by the mean value of the MVC when the experiment started and ended, respectively, compared with the Fourier fitting method. In the regression model, first, we set the initial strength as 100%. Thus, the initial timing was 0, *S* = *a* = 100. Then, the normalized strength after the repetitive lifting was obtained. Incorporating the time to obtain the MVC after the repetitive lifts and the mean normalized strengths of all subjects into the regression model, *b* can be derived.

## 3 Results

### 3.1 Comparison between the proposed method and maximal voluntary contraction (MVC) test

**FIGURE 6:**
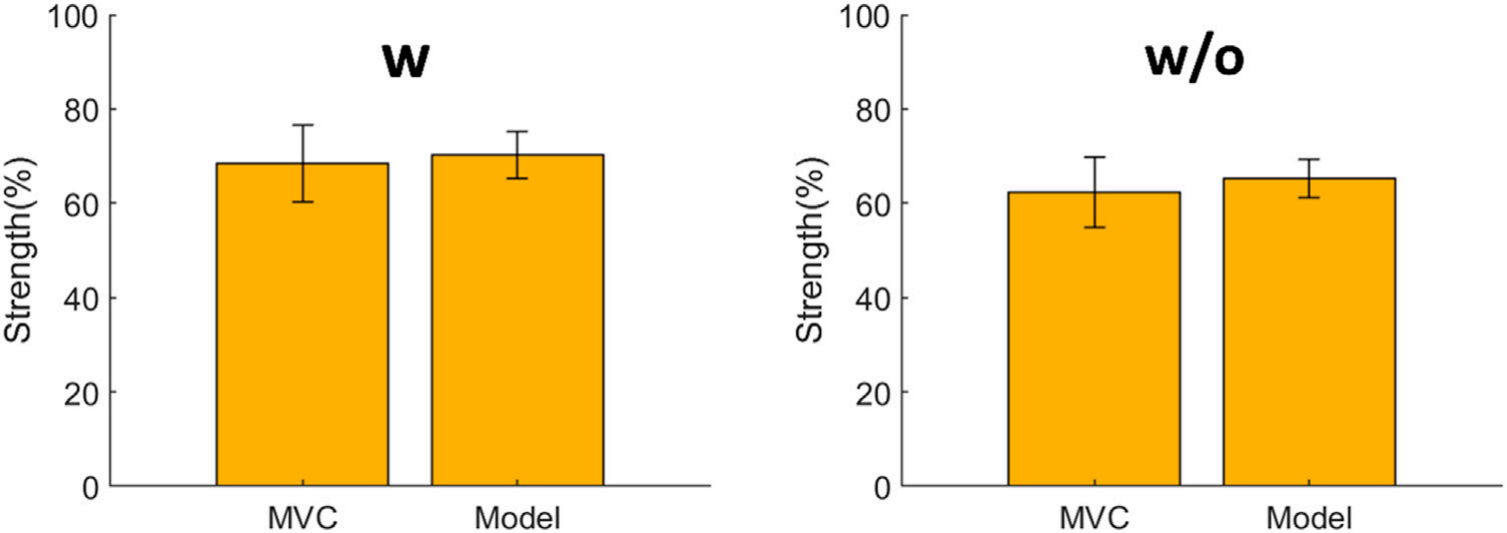
Comparison between the estimated lumbar strength using the proposed fatigue assessment method and the maximal voluntary contraction (MVC) obtained from the electromyography (EMG) test. No significant differences are observed under both conditions.

The sum of the joint strength levels using the fatigue assessment method was compared with the sum of the MVC of the left/right erector spinae and left/right multifidus among all the subjects (Figure 6). With or without the exoskeleton, no significant difference was observed between the lumbar strength estimated by the fatigue assessment method and the MVC. With the exoskeleton, the strength estimated by the model was 70% ± 5%, whereas the strength level of the MVC was 68% ± 8%. Without the exoskeleton, the strength estimated by the model was 65% ± 4%, whereas the strength level of the MVC was 62% ± 7%. The model estimated 2%–3% larger values than those with the MVC test.

### 3.2 Effect of the exoskeleton in the experimental task (body motion and lumbar load)

**FIGURE 7:**
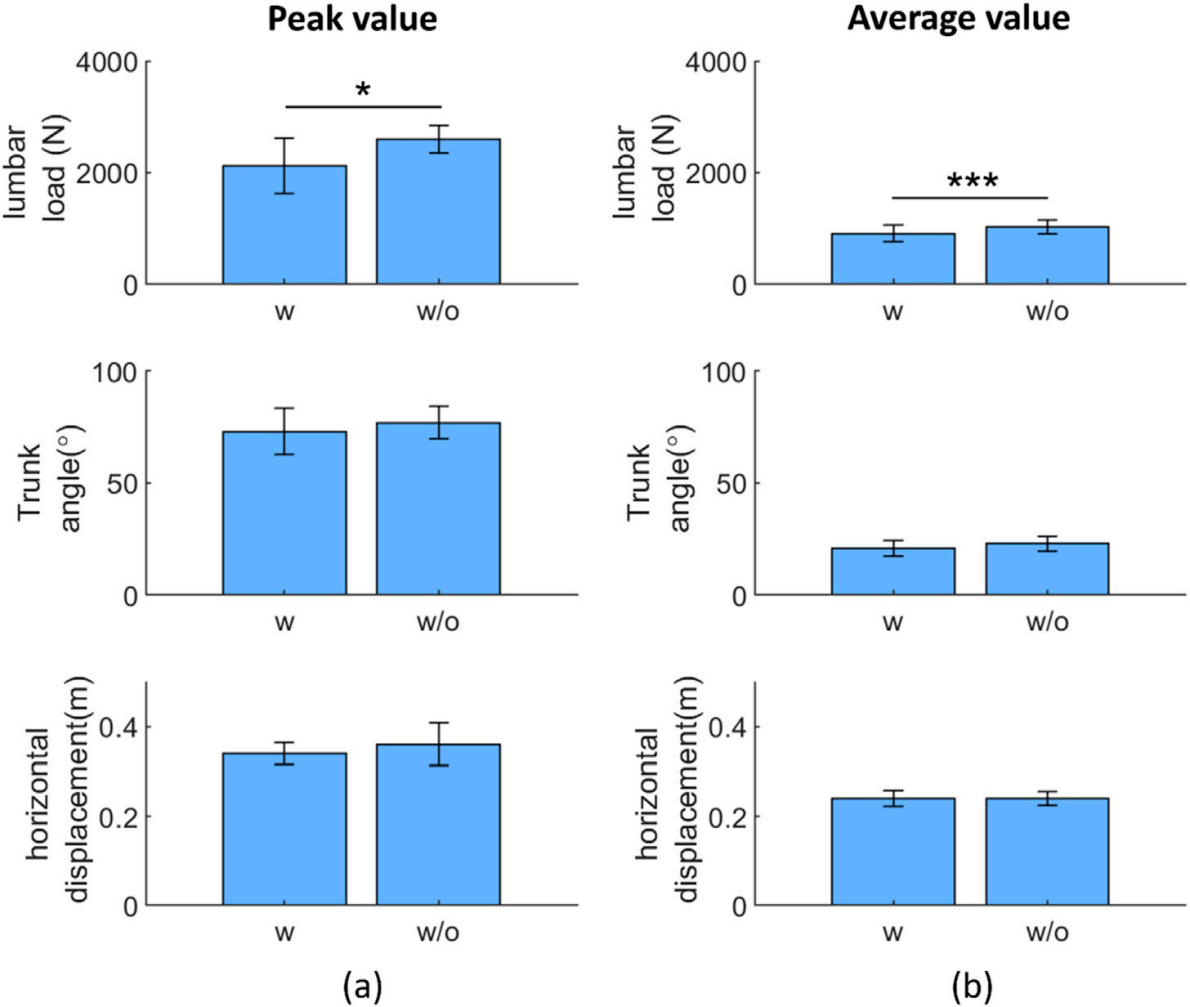
Paired t-test result: exoskeleton effect on the peak **(A)** and average **(B)** representative variables during repetitive tasks. Abbreviations: w, with; w/o, without (*p < 0.05; **p < 0.01; ***p < 0.005)

Figure 7 shows a comparison between the body motion parameters with and without the exoskeletons: the peak and average values of the lumbar load, trunk angle, and horizontal distance between the lumbar spine and wrist. For the lumbar load, the peak values were 2117.9 ± 492.4 N (w) and 2592.0 ± 243.1 N (w/o), and the average values were 901.6 ± 148.8 N (w) and 1015 ± 121.0 N (w/o), respectively. For the trunk angle, the peak values were 73.2° ± 10.4° (w) and 76.7° ± 7.3° (w/o), and the average values were 21.0° ± 3.6° (w) and 22.6° ± 3.2° (w/o), respectively. In ergonomic assessment, the maximal accepted load is related to the horizontal distance between the lumbar and wrist in manual lifting tasks. If the horizontal distance increases (decreases) when using the exoskeleton, the users may be less (more) willing to lift the load (Waters et al., 1993). For the horizontal distance between the lumbar and wrist, with and without assistance, the peak values were 0.35 ± 0.025 m (w), 0.36 ± 0.048 m (w/o), and the average trunk angles were 0.24 ± 0.017 m (w) and 0.24 ± 0.015 m (w/o), respectively. In comparisons with and without the exoskeleton, only the peak and average lumbar loads exhibited significant differences with *p*-values, and the corresponding test statistics (in the blank) were 0.024 (t(10) = −2.7) and <0.001 (t(10) = −5.3), respectively.

### 3.3 Effect on the extended motion (proposed method vs MVC regression method)

**FIGURE 8:**
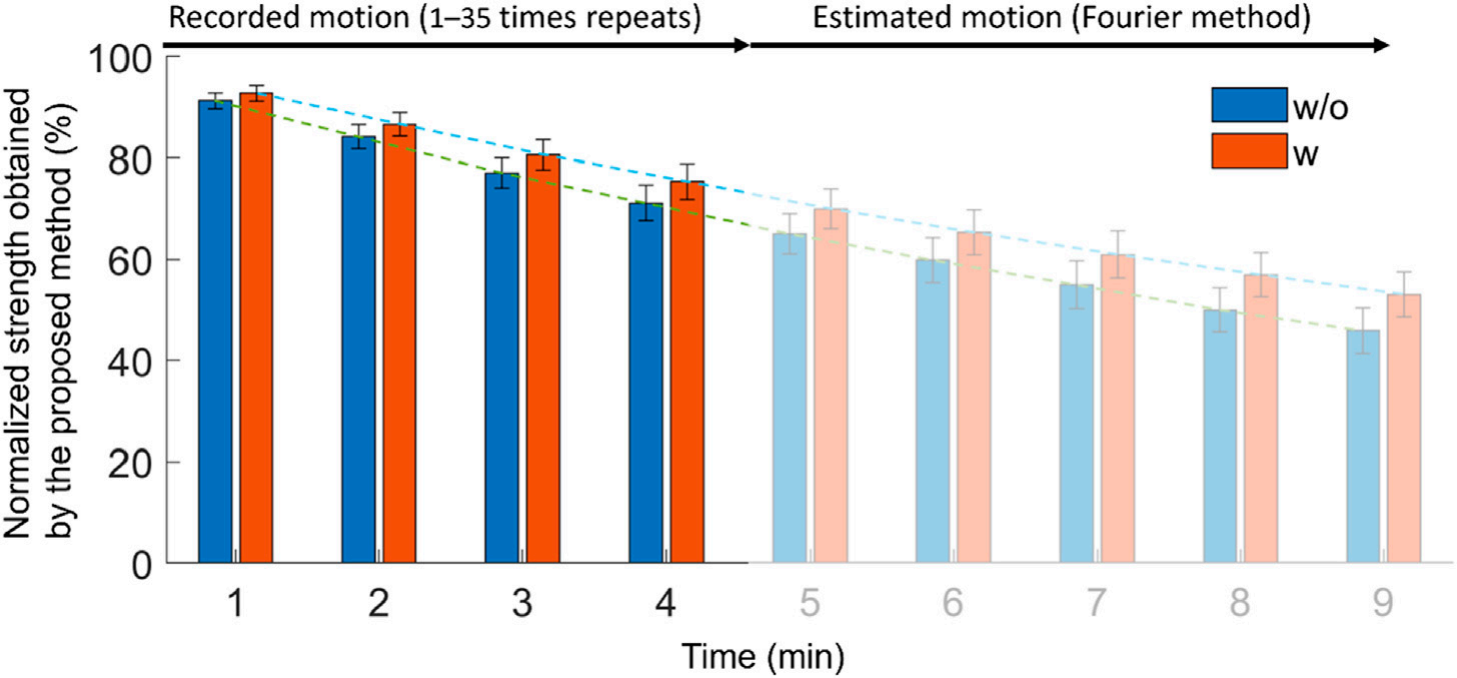
Effect of using exoskeleton on lumbar strength in the estimated motion by Fourier basis fitting procedure.

Figure 8 compares the lumbar strength with and without the exoskeleton using the Fourier basis function fitting method in the extended simulation motion. In the motion with and without the exoskeleton, the strength at 9 min decreased from 100% to 53.2% (w) and 46% (w/o). The strength between with and without conditions differed significantly (*p* < 0.05) from 1 to 9 min. The *p*-value and the corresponding test statistics (in the blank) from 1 to 9 min were 0.0019(t(10) = 4.2), 0.0012(t(10) = 4.5), <0.001(t(10) = 4.6), <0.001(t(10) = 4.7), 0.0011(t(10) = 4.5), <0.001(t(10) = 4.6), 0.001(t(10) = 4.6), <0.001(t(10) = 5.2), and <0.001(t(10) = 5.2), respectively. Using an exoskeleton helped the user to preserve strength from to 1–9 min by 1.5%, 2.5%, 2.5%, 4.2%, 5.0%, 5.5%, 6.0%, 6.9%, and 7.2%, respectively, compared with the condition without the exoskeleton.

**FIGURE 9:**
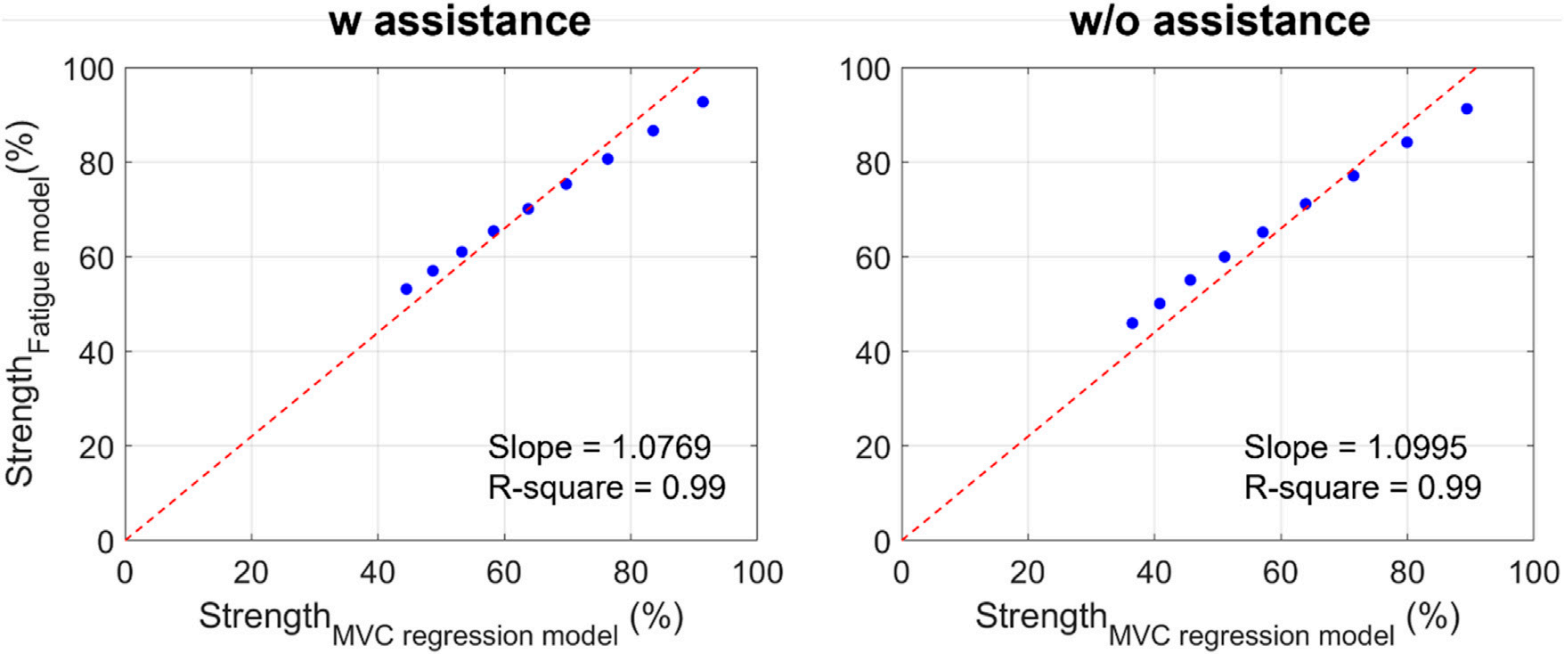
Comparison between the strength estimated by the MVC regression model and the proposed fatigue assessment method (red line: the linear regression line for blue dots without intercept; blue dots: the normalized strength obtained from the MVC regression model and from the proposed fatigue assessment model from 1 to 9 min).

Figure 9 shows the estimation of the Fourier method compared with that estimated by the regression model using the MVC data obtained in this study with and without the exoskeleton conditions. The regression model based on the MVC using the exoskeleton was $S=100e^{\left(-0.112t\right)}$ and $S=100e^{\left(-0.09t\right)}$ without the exoskeleton. The R-squares with and without the exoskeleton conditions were 0.99, and the slopes were 1.0769 for the exoskeleton condition and 1.0995 without the exoskeleton condition. The RMSE was 6.3 in the with-assistance and 7.5 without-assistance conditions and represents the normalized back strength.

## 4 Discussion

### 4.1 Comparison between the proposed method and MVC test

Usually, repetitive movements can be quantitatively assessed using physiological variables such as the heart rate or EMG (Godwin et al., 2009). In whole-body fatigue assessment, the fatigue level obtained from the fatigue assessment method has already been compared with the heart rate, which is similar (Yu et al., 2019). In this study, we focus on lumbar fatigue, and we compare the strength reduction after the task using the fatigue assessment method and MVC testing. The results in Figure 6 show that, compared with the MVC test, the fatigue assessment method provides a similar estimation of the lumbar strength with and without the exoskeleton. This implies that the proposed method can be used for lifting tasks. In addition, we consider that the slight difference between the MVC test and fatigue assessment method stems from the assumption of $S_{\max}$ in the fatigue model, which is the mean value of the maximal strength between 0° and 90° flexion (Chaffin et al., 2006). Based on the MVC test, the real strength assumption should be larger than $S_{\max}$ in this study.

### 4.2 Effect of the exoskeleton noted during the experimental task (body motion and lumbar load)

The difference in the greatest trunk angle was approximately 10% with and without the exoskeleton (standard deviation/mean value), which indicates that the users’ motion did not change significantly in these repetitive tasks, even when they experienced fatigue accumulation. In addition, it implies that the experimental data were in cyclic motion, and using the Fourier fitting procedure in the proposed fatigue assessment method was suitable for such motions, regardless of whether the exoskeleton was equipped or not.

In Figure 7, only the peak and average lumbar loads among all the biomechanical variables show a significant difference between using and not using exoskeletons in repetitive lifting, which are results similar to those obtained when testing other passive exoskeletons in both repetitive and non-repetitive lifting (Madinei et al., 2020) and for a previous testing of the same exoskeleton (Xiang et al., 2023). Although the trunk angles did not show significant differences with and without the passive type exoskeleton, it is difficult to draw the conclusion that the trunk angle was not limited by the exoskeleton in this study for two reasons: first, the result shows a difference between with and without exoskeleton conditions, even if it is not statistically significant; second, in previous studies, the limitation of the human trunk angle was revealed as they involved lifting tasks with active-type (Koopman et al., 2019) and passive-type exoskeletons (Baltrusch et al., 2018; Picchiotti et al., 2019).

Without restricting the motion, users can benefit from the above results by easily adapting and moving from one posture, task, or position to another while wearing exoskeletons, as expected from previous users (Omoniyi et al., 2020). However, not restricting the range of motion means that the exoskeleton cannot help users to reduce the lumbar load or fatigue by improving their lifting postures. For example, ISO 11228 suggests that people should adopt a squat posture to lift heavy masses instead of a stoop to reduce lumbar load (ISO 11228, 2021), and assistive devices could improve the users’ postures by reducing the maximal trunk angles.

Besides fatigue, other factors are important for the evaluation of the exoskeleton’s performance. Since the contact between the exoskeleton and the human body is complex when the user wears the lumbar-type exoskeleton, the exoskeleton affects not only the lumbar part but on other body segments. For example, for the overall body, the feeling of the subject to the exoskeleton is important, where satisfactory or subjective comfort can be scored by subjective investigations as a comprehensive assessment of a product (Baltrusch et al., 2018). Additionally, the friction between humans and the exoskeleton in motion will cause friction traumas. To avoid the occurrence of friction blisters, the permitted tangential traction can be presented with time history (Mao et al., 2017). Although more quantified analysis is required for these factors, it would be important to consider these factors together with the fatigue for the exoskeletons’ safety assessment in the future.

### 4.3 Effect noted in the extended motion (proposed method vs MVC regression method)

Figure 8 shows that using an exoskeleton can effectively reduce the lumbar fatigue from the first minute, and finally, at the ninth minute, the lumbar strength is 7.2% greater than that when not using the exoskeleton. This result implies that using an exoskeleton could effectively reduce lumbar fatigue. It is similar to the previous result that the passive exoskeleton could reduce the MVC by 9%–20% in 40 times lifting or flexion (Madinei et al., 2020).

In addition, Figure 9 shows that the correlation between the MVC regression model and fatigue assessment method is high (R-square = 0.99) with and without the exoskeleton. The MVC regression model is used for static conditions, such as holding the mass while maintaining a flexion posture. Thus, it can be inferred that the tendency of the strength change in symmetrical repetitive lifting tasks is similar to that observed in static conditions. However, the external loads acting on the hands are cyclically changed. This also indicates that we can simplify the movement and load from dynamic to static under special repetitive lifting conditions (without long-period resting and no significant posture change).

### 4.4 Advantages of the proposed method

Admittedly, the EMG method is mainstream and directly provides the change in muscle strength as well as individual differences in muscle strength. Alternatively, the proposed fatigue assessment method provides a relatively simple method for obtaining muscle fatigue, allowing us to conduct the ergonomics assessment in real-case scenarios such as agricultural fields, industrial factories, and rehabilitation centers. In addition, this method does not require users to have a strong technical expertise when conducting ergonomics assessments.

This method allows us to estimate the existing experiments and predict the subsequent movements. As shown in Figure 8, the motion from 5 to 9 min was predicted using the Fourier fitting equations. In this study, the lifting motions were assumed not to change with fatigue. However, Fuller et al. (2009) reported that the shoulder angle gradually decreased as the shoulder endured fatigue; hence, the current Fourier-based time-series analysis needs to be modified. For example, a linear equation can be added to the Fourier basis equations to simulate the increasing or decreasing shoulder angle.

The third advantage of this method is that it helps in the design process. Passive exoskeletons have a hysteresis effect on viscoelastic torque generation mechanisms, which leads to a higher assistive torque in flexion than in lifting (Madinei et al., 2020). Providing more assistive torque during the lifting phase could be a solution. Because there is no quantitative analysis of the hysteresis effect in exoskeletons, it is important to compare the characteristic curve of the assistive torque-related movements (e.g., trunk angle) and the relationship between the required lumbar torque (upper limit of assistive torque) and movement. As mentioned previously, our dynamic model can be used to estimate the relationship between the required lumbar torque and movement. The assessment method can predict the fatigue reduction during the necessary working period based on the characteristic curve of the assistive torque.

### 4.5 Limitations

We did not compare the differences between lifting and flexion with respect to the mass. Because both tasks exist in the same working scenario, it is necessary to consider them in plans. In addition, we did not account for the hysteresis effect in the exoskeleton model in this study, which should be addressed in the future. Additionally, how lumbar fatigue of using an exoskeleton is affected should be evaluated in the asymmetric tasks, which frequently occur in agriculture, rehabilitation, and industry scenarios.

The participants in this study were male adults aged 20–49 years. The number of elderly agricultural workers is increasing in aging societies, and their lumbar strength and motion patterns may differ from those of the youth. In the future, it will be necessary to focus on the elderly.

This study assessed only passive-type assistive devices. However, active exoskeletons may have a greater potential to reduce the physical load (De looze et al., 2016). An analysis of the active components is necessary.

A limitation of this study is related to the number of subjects recruited with similar and different body conditions, such as ages and genders. Ma et al. (2011) reported that the population characteristics and posture were external factors influencing fatigue resistance. However, how to quantify the influence of different factors on fatigue resistance remains unknown owing to the complexity of muscle physiology and the correlation among different factors. Therefore, more participants from different age groups, genders, and occupations will be investigated in the future. In addition, more participants with similar body characteristics will be included to enhance the robustness of the results. Moreover, human height, weight, muscular strength, and muscle mass, may also influence fatigue and should be conducted in a further investigation. For example, the BMI (body mass index) factor on fatigue was investigated for different weights and heights, and it was found that obese adults have greater fatigue than normal-weight adults in body trunk extension tests (Mehta and Cavuoto, 2017). Besides, it seems that smaller muscle strength or muscle mass will bring a longer endurance time, which can be explained as the lower absolute forces involving a lower muscle oxygen demand and, assuming a similar specific tension (Hicks et al., 2001).

Another limitation of this study is that it did not personalize the parameters *k* and $S_{\max}$ . The muscle conditions vary in different body characteristics, which may influence the final results. Therefore, the EMG method is considered suitable for investigating the basic performance of the muscles and the individual differences in muscle strength.

However, compared to the EMG method, the fatigue assessment model can provide a relatively simple and computationally efficient tool for measuring fatigue in virtual modeling. In addition, it allows ordinary people to do ergonomics assessments, whereas attaching EMG electrodes requires professional help.

## 5 Conclusion

A fatigue assessment method based on an exoskeleton’s characteristic curve and human dynamic simulation was used to assess the lumbar fatigue with and without an exoskeleton. Compared to EMG analysis, the fatigue assessment model can estimate fatigue in virtual modeling, allowing us to take the ergonomics assessment more easily in actual case scenarios. In the repetitive lifting experiment, the results estimated by this fatigue assessment method implied that the passive exoskeleton could effectively reduce lumbar fatigue and, therefore, could help reduce LBP. Furthermore, the tendency of the reduced strength estimated in the proposed assessment method is similar to that obtained from the EMG regression model in terms of the time history. These findings will contribute to the development of safer and more effective exoskeleton designs, ultimately enhancing the practical adoption of exoskeletons in various scenarios involving repetitive tasks.

## Data Availability

The raw data supporting the conclusions of this article will be made available by the authors, without undue reservation.
